# Supplementary material for: Therapeutic Applications of Mesenchymal Stem Cell Loaded with Gold Nanoparticles for Regenerative Medicine
Source: Pharmaceutics. 2023 Apr 30;15(5):1385. doi: 10.3390/pharmaceutics15051385 (PMC10222259; doi:10.3390/pharmaceutics15051385)
Supplement: Supplementary file 1 [file pharmaceutics-15-01385-s001.zip › pharmaceutics-2321099-supplementary.pdf]

pharmaceutics

MDPI

Supplementary Materials: Therapeutic Applications of Mesenchymal Stem Cell Loaded with Gold Nanoparticles for Regenerative Medicine

Wen-Yu Cheng, Meng-Yin Yang, Chun-An Yeh, Yi-Chin Yang, Kai-Bo Chang, Kai-Yuan Chen, Szu-Yuan Liu, Chien-Lun Tang, Chiung-Chyi Shen and Huey-Shan Hung

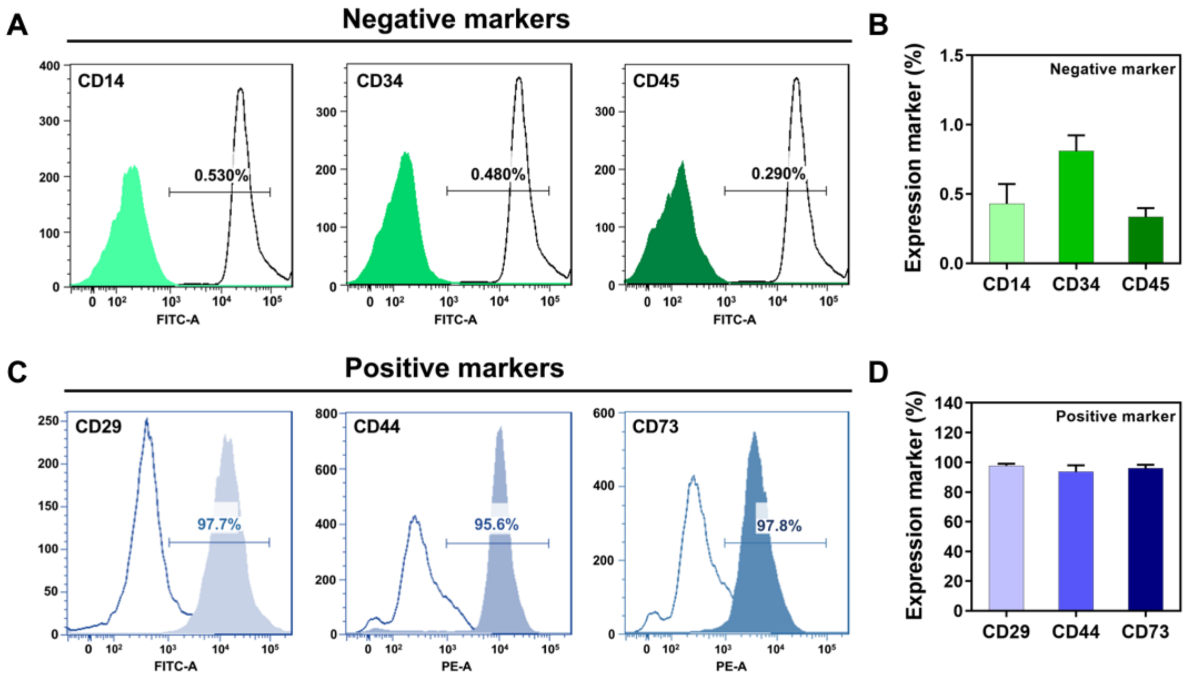

Figure S1. Phenotype identification of Wharton's jelly MSCs. The surface specific markers expression in cells were detected by flow cytometry. The expression of CD14, CD34 and CD45 negative markers were analyzed as 0.43%, 0.81 % and 0.335%. And the expression of CD29, CD44 and CD73 positive markers was 97.7%, 93.6% and 95.9% in cells, respectively. The above data represents of three independent experiments.

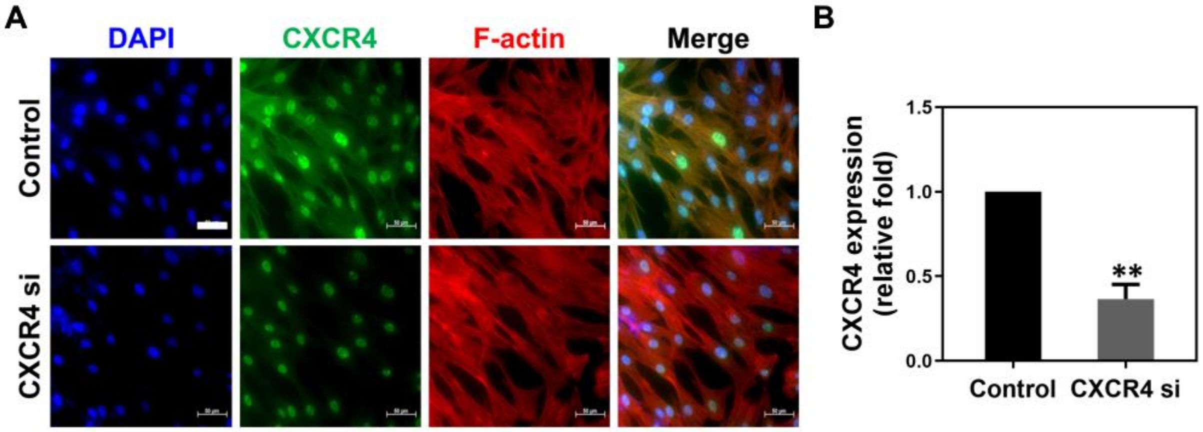

Figure S2. Knockdown of CXCR4 expression by CXCR4 siRNA transfection into MSC after 12 – 16 hours. (**A**) The expression of CXCR4 (green fluorescence) in MSC was observed by fluorescent microscopy. (**B**) The fluorescence intensity of CXCR4 was quantified, indicating the CXCR4 expression in MSC was indeed knocked down. Cell nuclei were stained by DAPI solution (blue fluorescence).

F-actin was displayed by rhodamine phalloidin (red fluorescence). The scale bars were set as 50 µm. $^{**}p < 0.01$: compared to the Control.

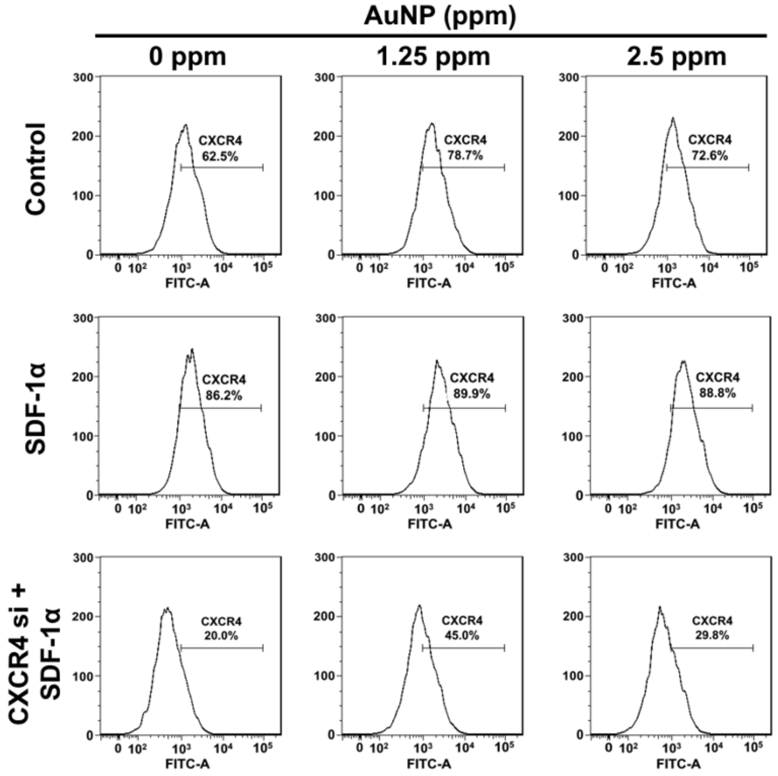

Figure S3. The histograms of CXCR4-positive MSCs detected by flow cytometry after treatments for 48 h.

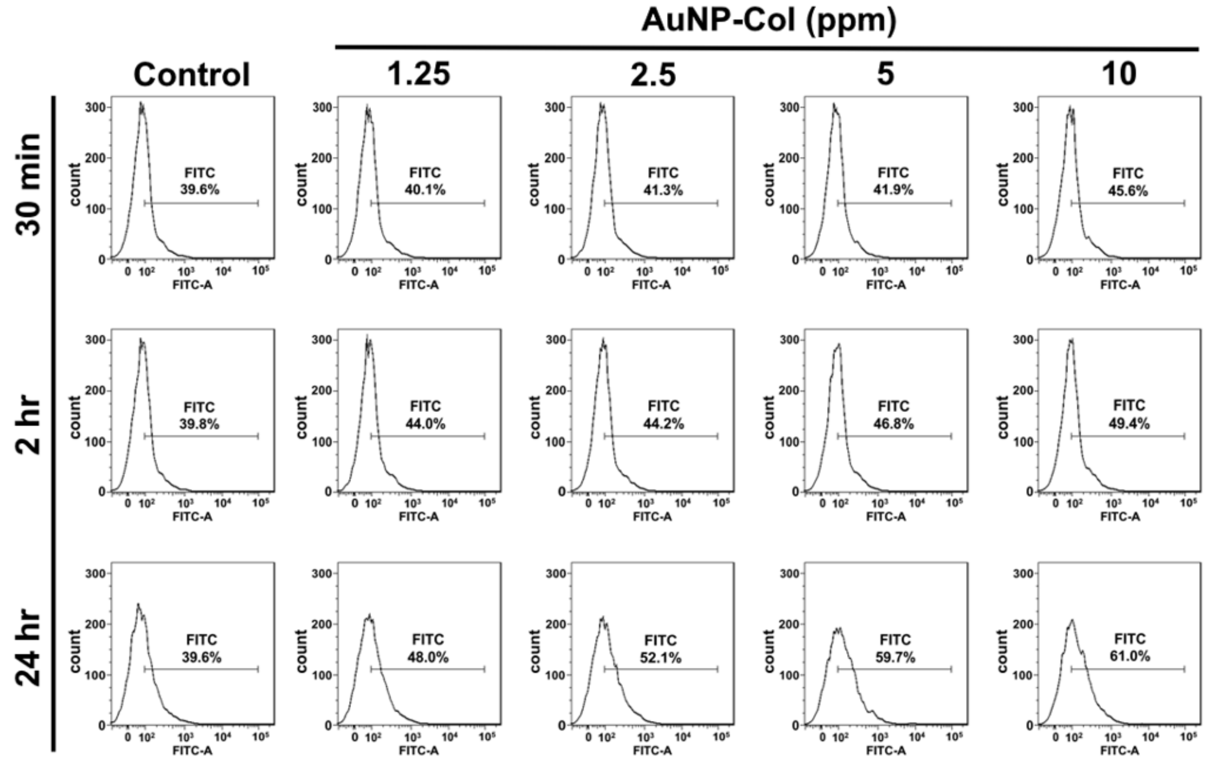

Figure S4. The histograms of AuNP-Col-FITC-positive MSCs detected by flow cytometry at 30 min, 2 h and 24 h are shown.

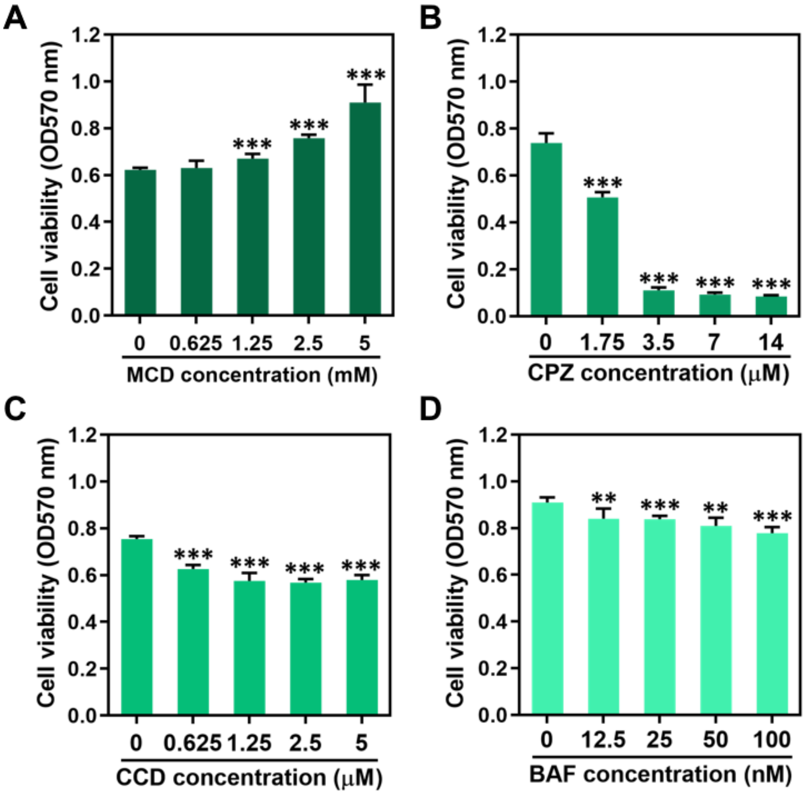

Figure S5. The cytotoxicity of various endocytosis inhibitors in MSCs was examined by MTT assay. The concentration of each inhibitor treated MSC was evaluated, and the appropriate concentrations were determined to be: (**A**) MCD: 2.5 mM, (**B**) CPZ: 2 μM, (**C**) CCD: 5 μM and (**D**) BAF: 100 nM. The results were quantified in triplicate. **$p < 0.01$, ***$p < 0.001$: compared to the Control (treatment without inhibitors).

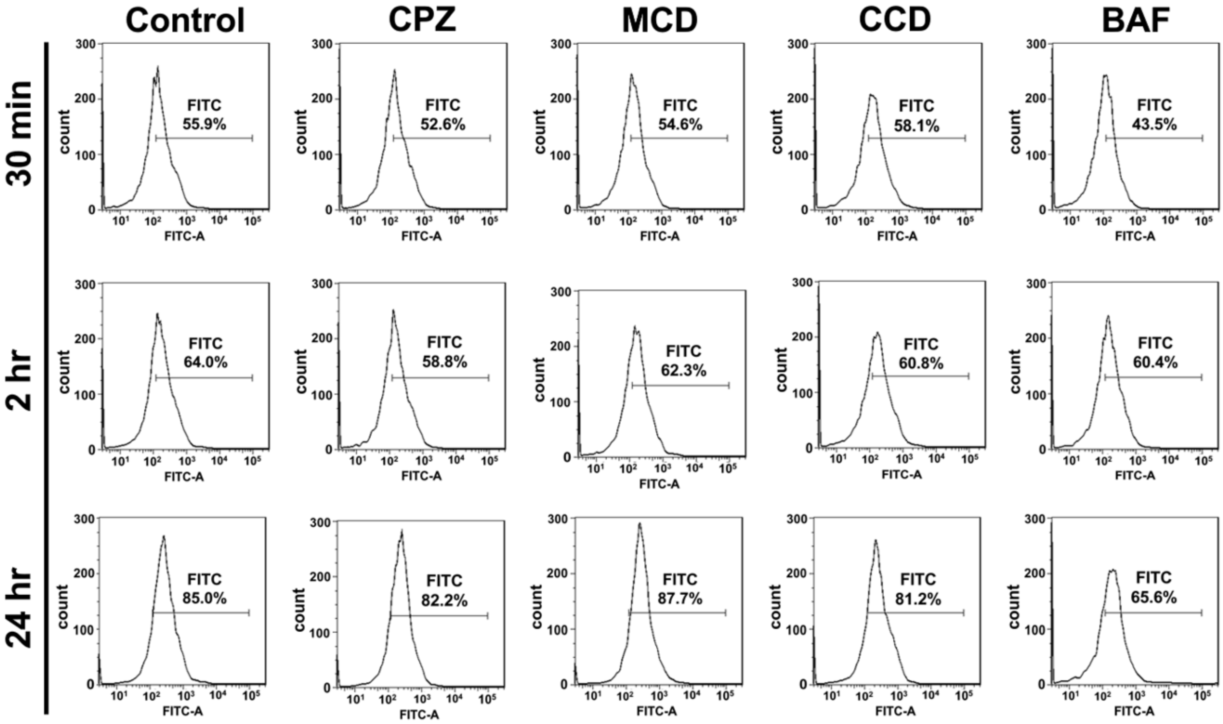

Figure S6. The histograms of AuNP-Col-FITC-positive MSCs detected by flow cytometry at 30 min, 2 h, and 24 h after treatment with various endocytosis inhibitors.
